# Circulating microRNA-1 in the diagnosis and predicting prognosis of patients with chest pain: a prospective cohort study

**DOI:** 10.1186/s12872-018-0987-x

**Published:** 2019-01-05

**Authors:** Tong Su, Xiaonan Shao, Xiaopu Zhang, Zhijun Han, Chengjian Yang, Xun Li

**Affiliations:** 1 Department of Cardiology, The First Affiliated Hospital of Soochow University, Suzhou, 215006 Jiangsu China; 2 Department of Cardiology, The Third Affiliated Hospital of Soochow University, Changzhou, 213003 Jiangsu China; 3 Department of Nuclear Medicine, The Third Affiliated Hospital of Soochow University, Changzhou, 213003 Jiangsu China; 4 Department of Neurology, The Third People’s Hospital of Changzhou, Changzhou, 213001 Jiangsu China; 5 Department of Cardiology, The Second People’s Hospital of Wuxi Affiliated to Nanjing Medical University, Wuxi, 214000 Jiangsu China

**Keywords:** Acute myocardial infarction, Chest pain, MicroRNA-1, Cardiac troponin, Biomarker

## Abstract

**Background:**

To investigate the early diagnostic and prognostic value of microRNA-1 in patients with acute chest pain.

**Methods:**

This study enrolled 341 patients attacked by chest pain within 3 h, and another 100 volunteers as control group. Circulating microRNA-1 was collected and determined by real-time quantitative reverse transcription-polymerase chain reaction. The clinical follow-up period was 720 days.

**Results:**

There were 174 patients in acute myocardial infarction (AMI) group, 167 in non-AMI group. The relative expression of microRNA-1 was significantly increased within 3 h in AMI group, and it continued rising within 12 h, lower at 24 h than that 12 h in AMI group without reperfusion therapy. Otherwise, microRNA-1 concentration was markedly low at 12 h after primary percutaneous coronary intervention in AMI group. The 95% reference range of circulating microRNA-1 was 0.171–0.653. It was significantly available for microRNA-1 to early diagnose AMI with an optimal cutoff value of 2.215 and diagnostic accuracy could be improved when combined with cardiac troponin I. It was not statistically significant for microRNA-1 to forecast future AMI but might prognose mortality of 720 days in chest pain patients. In patients with chest pain, microRNA-1 concentration was high with major adverse cardiac events within 30 days, low with high overall survival within 720 days.

**Conclusions:**

Circulating microRNA-1 might diagnose early AMI and predict the prognosis of patients with chest pain.

## Background

Acute myocardial infarction (AMI) is a severe cardiac disease with high mortality and poor prognosis, and acute chest pain (ACP) causes hospital admission worldwide. It is critically significant to differentially diagnose whether patients with ACP are suffering from AMI or not without delay [1]. Firstly, the rapid and accurate diagnosis of AMI is critical for the initiation of effective evidence-based medical management and treatment [2–4]. Secondly, there is a potential to enhance the efficiency of emergency department (ED) by rapidly and reliably rule-out AMI, which also reduce patients’ anxiety and rational managing medical resources [5, 6]. At present, atypical AMI have occurred frequently, resulting in difficult diagnosis and delayed treatment. Accordingly, sometimes we have to combine with Cardiac troponin (cTn), a representative of myocardial enzyme, to make a correct diagnosis. However, cTn usually begins to rise about 3 h after the onset of AMI, reaches a peak around 24 h, and increases in patients with aortic dissection (AD), end-stage renal damage (ESRD), pulmonary embolism (PE) and acute heart failure (AHF) [7, 8]. If there is a contraindication of coronary angiography (CAG) or patients and their families refuse to undergo invasive treatment at this time, a diagnostic and therapeutic dilemma will occur [9].

MicroRNAs (MiRs) are small RNAs that regulate expression of proteins by acting on the 3’untranslated region (UTR) of target genes encoding these proteins [10–12]. They participate in the regulation of a variety of signal pathways and maintain the homeostasis of the body environment, and there is a significant difference in the expression profiles between healthy and various disease patients, while being stable in the peripheral blood and having the potential for diagnosis and treatment of AMI [13]. MicroRNA-1 (MiR-1) is one of miRs which is expressed in cardiomyocytes, and it is the first miR found to be associated with cardiovascular development [14]. Moreover, some studies suggest that miR-1 directly participates in the regulation of the whole pathological process of AMI and AMI prognosis can be optimized by detecting the expression of miR-1 and upstream regulation [15–17].

## Methods

### Materials and study design

A prospective cohort study was performed on all patients with ACP who were admitted to ED of our center from November 2012 to December 2015. This study enrolled 341 patients attacked by chest pain within 3 h, and additional 100 volunteers as control group. Exclusion criteria: ①Patients complained of onset time > 3 h. ②ESRD or cancer patients. ③ACP caused by trauma, drugs or medical intervention. Whole venous blood samples (3–5 mL) were collected as soon as patients arrived at ED, and then EDTA-K2 anticoagulation, centrifuged plasma, stored at − 80 °C for testing.

This study was in line with the medical ethics standards and the consent of the hospital ethics committee. All patients signed the informed consent.

### Adjudicated final diagnosis

Diagnostic criteria of AMI and cTn levels were defined as recommended in the current guideline [4, 18]. In brief, AMI was diagnosed when there was evidence of myocardial necrosis in association with a clinical setting consistent with myocardial ischemia. Myocardial necrosis was diagnosed by at least one cTn value above 99th percentile together with a significant rising or falling [19, 20]. The final diagnosis was adjudicated by two independent cardiologists, in case when disagreement occurred, cases were reviewed and adjudicated in conjunction with the third cardiologist. According to the final diagnosis, patients with ACP were divided into AMI-group and non-AMI group. Those individuals who received physical examination in the same period were taken as non-ACP control group.

### Determination of circulating miR-1

Extraction of total RNA from plasma samples with mirVana PARIS kit (Applied Biosystem, USA). Reverse transcription of miRs using miScript reverse transcription kit (Applied Biosystem, USA). According to manufacturer’s protocol (Applied BioSystems, USA), miR-1 was quantitated by using TaqMan (Applied Biosystem, USA) miR quantitative reverse transcriptase-polymerase chain reaction (qRT-PCR) assay, and U6 RNA was performed as a miR internal control described previously [21]. All assays were performed in triplicate. Data were analyzed with automatic setting for assigning baseline. The threshold cycle (Ct) was defined as the fractional cycle number at which the fluorescence exceeded the given threshold. The plasma levels of miR were performed and analyzed independently by two investigators who were blinded to the clinical characteristics of patients. The relative expression of the miRs were calculated using the 2^-ΔΔCt^ method according to the well-established methods [22].

### Investigational cTnI measurements

Levels of serum cTnI was measured with BECKMAN DXI800 system and followed by the manufacturer’s instructions. The normal reference range of upper limit was 0.01 μg/L. We took the first test result for statistics.

### Follow-up and clinical end-points

Patients were followed-up at 3, 12, and 24 months after discharging by telephone calls. The co-primary prognostic end points were overall survival after 720 days. The secondary prognostic end point was major adverse cardiac events (MACEs), defined as the composite of all-cause mortality (AMI (including index event), cardiogenic shock, ventricular tachyarrhythmias, or higher degree atrioventricular block at 30 days).

### Statistical analysis

Categorical variables were presented as numbers and percentage, and continuous variables as median and interquartile range (IQR). Continuous variables were compared using the Kruskal-Wallis H test among the three groups, and the Mann-Whitney-U test between the two groups. Categorical variables were used with the Pearson χ2 test or Fisher’s exact test as appropriate. Two-way repeated measures anova was used to compare the differential relative expression of miR in two groups at different time points. Receiver operating characteristic curve (ROC) was constructed to assess the diagnostic efficacy of circulating miR-1 and cTnI. The area under the ROC curve (AUC) was calculated, and when AUC < 0.5 means no diagnostic value [23]. Kaplan–Meier survival analysis was performed with log-rank values to assess statistical significance. Statistical analyses were performed with the SPSS 19.0 (Inc., an IBM company, USA) statistical package. All hypothesis testing was two-tailed. A value *P* < 0.05 was considered statistically significant.

## Results

### Characteristics of patients

Baseline characteristics of three groups were shown in Table 1. There were 174 patients in AMI group, 167 in non-AMI group, including 70 cases of stable angina, 44 cases of unstable angina, 34 cases of heart failure, 5 cases of peptic ulcer, 4 cases of myocarditis, 2 cases of pericarditis, 2 cases of pulmonary embolism, 2 cases of myocardial bridge, 2 cases of aortic dissection, 1 case of penetrating abdominal aortic ulcer and 1 case of pneumonia. Compared with the non-ACP group, there was no significant difference in gender, hypertriglyceridemia, history of CVD, heart rate and body mass index (BMI) in non-AMI group, and gender, hypercholesterolemia, history of CVD in AMI group. Compared with non-AMI group, the AMI group had no significant difference in age, gender, hypertension, diabetes, hypercholesterolemia and history of CVD.

**Table 1:** Baseline characteristics of patients in each group

Group	AMI	non-AMI	non-ACP	χ^2^/ F / Z value	*P* value	P_1_ value	P_2_ value	P_3_ value
	(*n* = 174)	(*n* = 167)	(*n* = 100)					
Age(years)	68 (55, 75)	67 (60, 75)	56 (45, 69)	26.894	0.000	0.252	0.000	0.000
Male gender	121 (69.54)	121 (72.46)	75 (75)	1.536	0.464	0.460	0.226	0.566
Current smokers	116 (66.67)	47 (28.14)	8 (8)	126.207	0.000	0.000	0.000	0.000
Hypertension	127 (72.99)	119 (71.26)	30 (30)	85.888	0.000	0.999	0.000	0.000
Diabetes mellitus	53 (30.46)	45 (26.95)	6 (6)	32.261	0.000	0.259	0.000	0.000
Hypercholesterolemia	14 (8.05)	15 (8.98)	3 (3)	5.585	0.061	0.382	0.079	0.018
Hypertriglyceridemia	36 (20.69)	31 (18.56)	10 (10)	9.999	0.007	0.043	0.003	0.187
History of CVD	7 (4.02)	7 (4.19)	5 (5)	0.139	0.933	0.890	0.710	0.804
Heart rate(bpm)	88.5 (73, 107)	72 (64, 81)	72 (61, 81)	97.883	0.000	0.000	0.000	0.776
Body mass index(kg/m^2^)	24.15 (21.51, 27.41)	23.62 (20.43, 26.03)	23.1 (20.46, 25.78)	6.861	0.032	0.024	0.032	0.773
Creatinine(μmol/l)	88.4 (70.65, 109.25)	70.9 (65.2, 89.3)	71 (63.7, 85.3)	38.343	0.000	0.000	0.000	0.298
GFR (mL/min/1.73 m^2^)	71.46 (52.68, 90.3)	87.3 (73.37, 96.21)	93.54 (76.26, 105.61)	45.125	0.000	0.000	0.000	0.002
Creatine kinase MB(U/L)	33 (14.5, 104.2)	15.5 (12.3, 19.5)	8.9 (8.3, 9.45)	185.337	0.000	0.000	0.000	0.000
cTnI(μg/L)	3.23 (0.62, 16.62)	0.05 (0.00, 0.34)	0.00 (0.00, 0.00)	233.657	0.000	0.000	0.000	0.000

Categorical variables were presented as numbers (%), continuous variables were presented as median and interquartile range (IQR). Continuous variables were compared with the Kruskal-Wallis H test of the three groups, and with the Mann-Whitney-U test between the two groups. Categorical variables were used with the Pearson χ2 test or Fisher’s exact test as appropriate. *ACP* acute chest pain, *AMI* Acute myocardial infarction, *CVD* cardiovascular disease, *GFR(CKD-EPI)* glomerular filtration rate (using the chronic kidney disease epidemiology collaboration formula based on plasma creatinine levels obtained at presentation in the Emergency Department), *cTnI* cardiac troponin I. χ2/ F / Z values were for the comparison of three groups. P for the comparison within three groups, P_1_ for the comparison between AMI group and non-AMI group while P_2_ for the comparison between AMI group and non-ACP group, P_3_ for the comparison between non-AMI group and non-ACP group

### The relative expression of miR-1 was different in each group

**Fig. 1:**
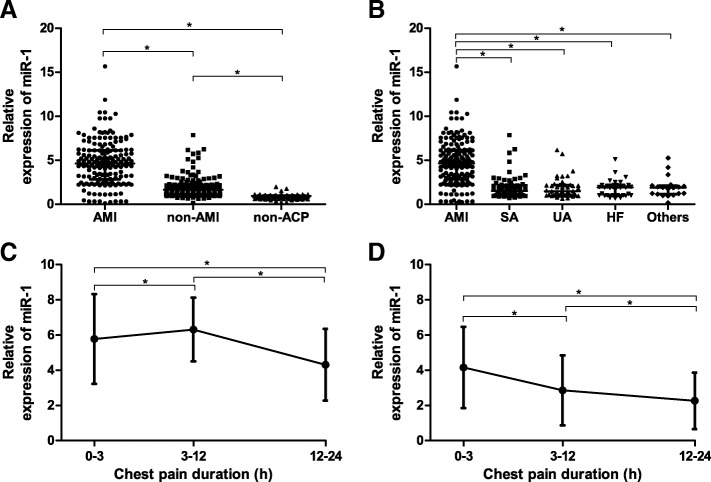
The plasma concentrations of miR-1 in AMI group, non-AMI group and non-ACP group (**a**). The levels of plasma miR-1 in subgroups of the non-AMI patients (**b**). The changes in different time of miR-1 concentrations in patients without reperfusion therapy (**c**) and with primary percutaneous coronary intervention in AMI group (**d**). * = *P* < 0.001

The relative expression of circulating miR-1 in AMI group and non-AMI group were markedly higher than that of the control group, and AMI group was higher than non-AMI group. Furthermore, levels of miR-1 were significantly elevated in AMI patients than any other subgroup of the non-AMI patients (all *P* < 0.001, Fig. 1).

### Reperfusion therapy affected miR-1

According to the AMI diagnosis guideline, the concentration of miR-1 could be detected at the late stage since the symptom broke out. Additionally, miR-1 concentration at 12 h was markedly low after primary percutaneous coronary intervention (PPCI) in AMI group (all *P* < 0.001, Fig. 1).

### Diagnostic value of miR-1 in patients with ACP

**Fig. 2:**
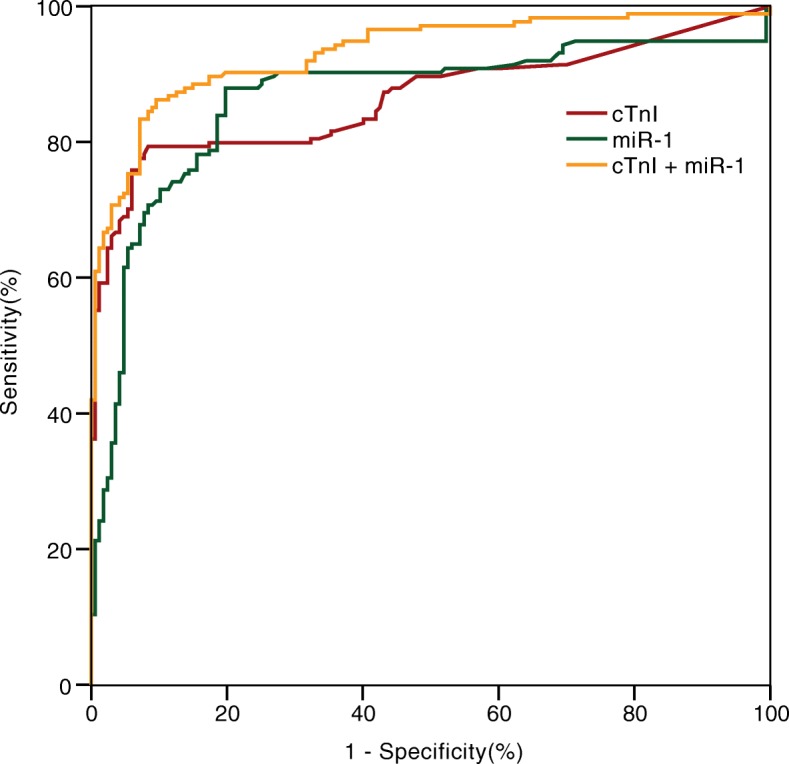
Receiver operating characteristic curves showed diagnostic accuracy of circulating miR-1 (< 3 h) in comparison and combination with cTnI in the early AMI

ROC curve analysis showed that miR-1 has the potential for early diagnosis of AMI. The diagnostic efficacy was comparable with cTnI, and the combination of them could provide more diagnostic information (Fig. 2**,** Table 2).

**Table 2:** Diagnostic performance of miR-1, cTnI and combination in patients with early AMI

Biomarker	AUC (95%CI)	P value	Sen.	Spe.	PPV	NPV	LR+	LR-
miR-1	0.863 (0.820~0.906)	0.000	87.9	80.2	0.823	0.865	4.450	0.150
cTnI	0.864 (0.823~0.906)	0.000	79.3	91.6	0.908	0.810	9.461	0.226
miR-1 + cTnI	0.931 (0.904~0.959)	0.000	86.2	90.4	*0.799*	*0.947*	*3.816*	*0.005*

*miR-1*microRNA-1, *cTnI* Cardiac troponin I, *AUC* the area under the receiver operating characteristics curve, *Cl* confidence interval, *Sen.* sensitivity (%), *Spe.* specificity (%), *PPV* positive predictive value, *NPV* negative predictive value, *LR+* positive likelihood ratio, *LR-* negative likelihood ratio; Italics mean that joint diagnosis is calculated by parallel test method

### The 95% reference range of miR-1

The sample data of non-ACP patients were verified to be normal distribution by Shapiro-Wilk test (*P* > 0.05). According to the formula ($\overline{x}$ ± μ × s, μ = 1.96), the 95% reference range of plasma miR-1 in non-ACP patients was 0.171–0.653.

### Prognostic value of miR-1 in patients with ACP

**Fig. 3:**
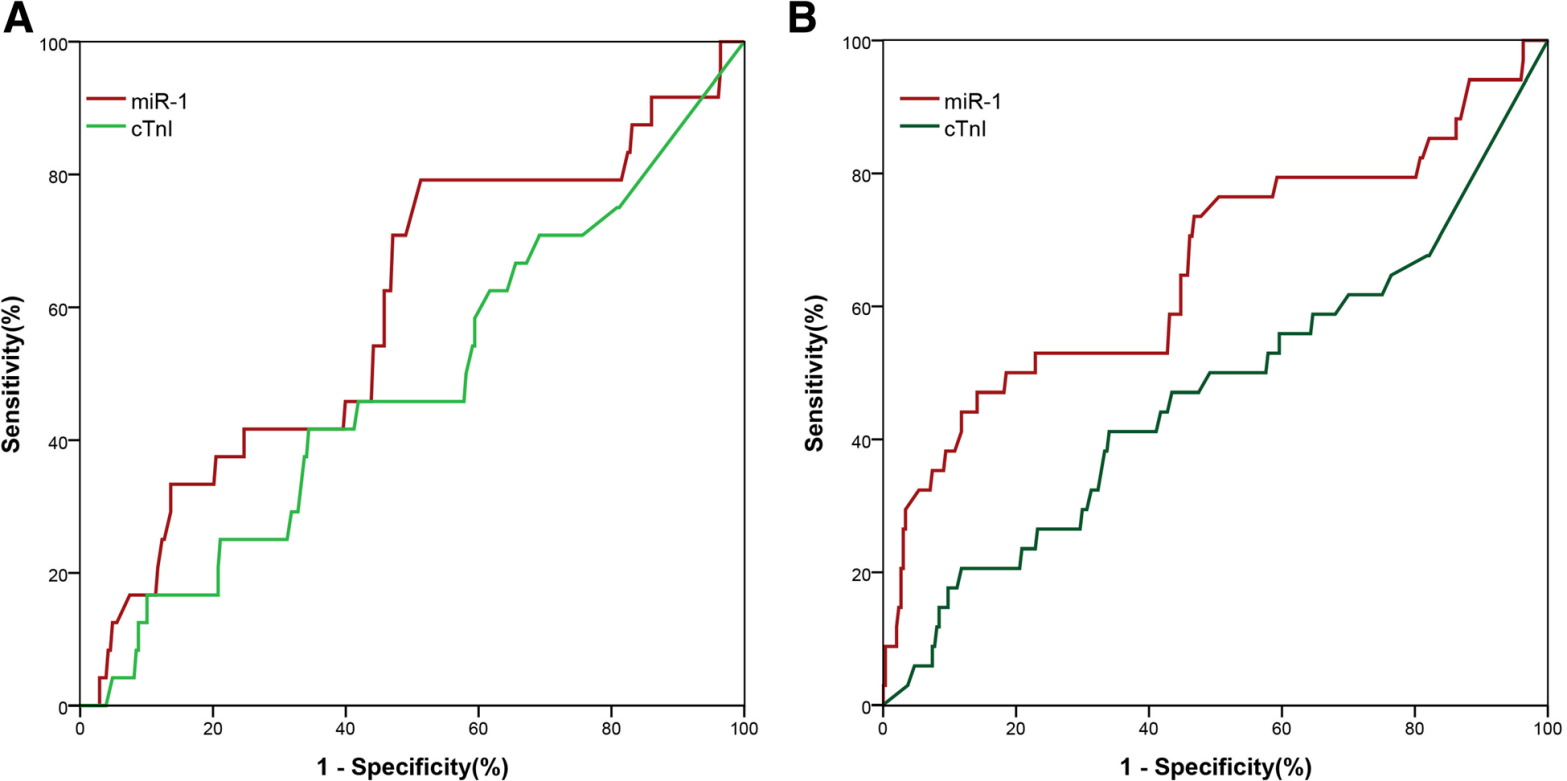
Receiver operating characteristic curves showed prognostic performance of MACEs (30 days) of miR-1 (< 3 h), cTnI in patients with chest pain (**a**). Receiver operating characteristic curves showed prognostic performance of overall mortality (720 days) of miR-1, cTnI in patients with chest pain (**b**)

**Fig. 4:**
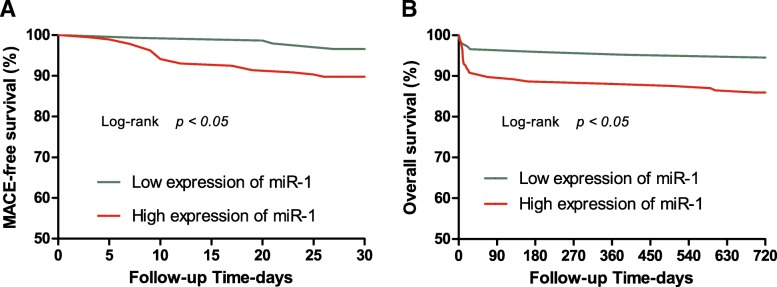
The prognostic accuracy of miR-1 (< 3 h) to predict short-term (30 days) MACE-free survival (**a**); The prognostic accuracy of miR-1 to predict long-term overall survival (720 days) in chest pain patients (**b**). Kaplan Meier survival curves for all patients with chest pain manifesting with cumulative survival during short-term (30 days) and long-term (720 days) follow-up. Patients were subdivided into low expression of miR-1 and high expression of miR-1 according to optimal cut-off value (2.215) calculated by the Youden index (sensitivity 87.9%, specificity 86.9%). Log-rank values were employed to evaluate statistical significance (*P* < 0.01)

Median follow-up time was 49 months (IQR 27, 57 months), there were 34 (9.97%) deaths with a median time to death of 18 days (IQR 9, 168 days). All patients with ACP were included in the follow-up. MiR-1 was not statistically significant to predict future AMI in ACP group (supplementary material for review). The prognostic accuracy of miR-1 and cTnI for short-term MACEs (30 days) and long-term mortality (720 days) in ACP patients by ROC curve analysis were shown in Fig. 3, Table 3. Only in the case of miR-1 was there a borderline significant AUC for short-term MACEs and long-term mortality. According to Kaplan–Meier analysis (only shown for miR-1, which displayed the better performance in ROC curve analysis), the survival outcomes of patients with ACP in both groups were significantly better in miR-1 with relatively low concentrations (≤2.215), as shown in Fig. 4 (both *P* < 0.05).

**Table 3:** Prognostic value of miR-1 and cTnI in patients with ACP

Prognostic performance for MACEs (30 days) in patients with ACP								
Biomarker	AUC (95%CI)	*P* value	Sen.	Spe.	PPV	NPV	LR+	LR-
miR-1	0.620 (0.500~0.740)	0.046	80.0	50.3	0.113	0.970	1.610	0.397
cTnI	0.469 (0.345~0.594)	0.612	40.0	66.5	0.086	0.933	1.192	0.903
Prognostic performance for overall mortality (720 days) in patients with ACP								
Biomarker	AUC (95%CI)	*P* value	Sen.	Spe.	PPV	NPV	LR+	LR-
miR-1	0.666 (0.555~0.778)	0.001	47.1	86.3	0.276	0.936	3.440	0.613
cTnI	0.474 (0.359~0.589)	0.614	20.1	88.6	0.167	0.910	1.806	0.896

*AUC* the area under the receiver operating characteristics curve, *ACP* acute chest pain, *miR-1* microRNA-1, *cTnI* Cardiac troponin I, *Cl* confidence interval, *Sen.* sensitivity (%), *Spe.* specificity (%), *PPV* positive predictive value, *NPV* negative predictive value, *LR+* positive likelihood ratio, *LR-* negative likelihood ratio

## Discussion

In this prospective cohort study of 341 patients within 3 h after the onset of ACP and 100 non-ACP patients, we evaluated the diagnostic and prognostic value of plasma levels of miR-1. This study suggested that circulating miR-1 levels in patients with early AMI were significantly higher than those non-AMI patients and controls, consistent with the findings of some studies [21, 24–26]. The study had demonstrated that a timely revascularization treatment within 3 h after AMI would repair the ischemic myocardium and ultimately reduce the mortality to get an optimistic prognosis [27]. Hence, the aim of this study was to evaluate the early diagnostic and prognostic value of miR-1 in patients with AMI, including patients whose symptom onset time was < 3 h, and no reperfusion treatment was performed before the sample was taken in the way to avoid affecting the outcomes. Although this study showed that the diagnostic efficacy of miR-1 on AMI is comparable to that of cTnI, combined diagnosis could reduce the risk of delaying in missed diagnosis. Based on the current studies of the early diagnosis of AMI in circulating miR series, we found most evidences of miR-1, miR-499 and miR-208b. Devaux Y et al. [28] recruited 1155 suspected of AMI patients and eventually diagnosed with 224 cases AMI. Although diagnosed patients had significantly elevated miR-499 and miR-208b, their diagnostic efficacy was no better than cTnI, even combined diagnosed with miR-499 and miR-208b. Furthermore, miR-499 and miR-208b did not show any advantage in predicting the prognosis of AMI patients for 2 years follow-up. Widera C et al. [29] suggested that in the differential diagnosis of AMI and UA, miR-499, miR-208b did not show significant differences. Researches showed that miR-1 was the most abundantly expressed miR in cardiomyocytes and played an important role in maintaining normal physiological functions of cardiomyocytes, such as regulation of endothelial function, angiogenesis, and apoptosis [25]. The study established animal models to compare the expression of miR-1 in drug-induced secondary myocardial ischemia and myocardial infarction, suggested that miR-1 might be superior in the diagnosis of myocardial infarction to myocardial injury in animal model [30]. So far, the benefit of miRs in prognostic value was not well studied as the potential diagnostic benefit in cardiovascular medicine. Ai J et al. [31] manifested that increased miR-1 had prompted the role of myocardial contractility and diastolic dysfunction after AMI. Cheng Y et al. [25] indicated that elevated circulating miR-1 can predict ventricular arrhythmias after AMI. Qipshidze Kelm N et al. [32] designed a mouse model of AMI which was established by ligation of the anterior descending coronary artery of mice with different ages, and the levels of circulating miR-1 and miR-133a were measured. This study suggested that miR-1 elevation was more pronounced, and increased circulating miR-1 levels in older mice are positively associated with increased heart dysfunction after AMI. Further clinical trial found that the levels of circulating miR-1 after AMI significantly correlated with absolute change in infarct volume and showed a trend for correlation with absolute change of left ventricular ejection fraction [17]. These researches provided the basis for miR-1 to evaluate the prognosis of AMI patients. In our study, miR-1 can predict the prognosis of ACP patients, which may be attributed to its diagnostic value in the early occurrence of AMI in ACP patients. In addition, there are several points need to be clarified in this study. First, cTnI testing results was not blind to clinician, which was susceptible to become the subjective factors of clinicians, but miR-1 made the results more objective. Second, the adjudication of AMI was performed by using the criteria by universal definition, putting cTn in a decisive position. Therefore, it was inevitable to cause test bias leading to embarrassment of new biomarker to be superior to cTn. Third, our study suggested that the sensitivity of cTnI was relatively low when AMI onset was within 3 h, combined with miR-1 parallel test method could improve the sensitivity of the diagnosis at this time (sensitivity 95.98%, specificity 74.85%). Fourth, there were many reports that miR-1 had a relationship with tumor at present, thus this study excluded patients with cancer to avoid bias [33]. Fifth, no conclusions could be made about patients with ESRD as they were excluded from the study. Of note, the concentrations of miR-499 and cTn were identified increasing in patients with ESRD without any evidence of cardiac injury [34]. Sixth, this study was inevitably affected by the sample size and by individual differences, complications, conditions, clinical time in different patients, which might cause the standard deviations of the data dispersed. Further study of the role of circulating miRs in the diagnosis and prognosis of AMI needs to improve and enrich the AMI evaluation system, in conjunction with the global acute coronary event registration score (GRACE) and emergency medical score (REMS), which makes greater sense for clinical medicine. The usage of miRs in clinical applications of AMI still requires large-scale, multi-center, registration studies to validate.

## Conclusion

Our study suggested that circulating miR-1 might diagnose early AMI in patients with ACP, which was almost equal to cTnI, and diagnostic accuracy could be elevated when combined miR-1 with cTnI. Furthermore, miR-1 might play a role in predicting the prognosis of patients with suspected AMI.

## Abbreviations

ACP: Acute chest pain
AD: Aortic dissection
AHF: Acute heart failure
AMI: Acute myocardial infarction
AUC: Area under the ROC curve
Cl: Confidence interval
Ct: Threshold cycle
cTn: Cardiac troponin
cTnI: Cardiac troponin I
CVD: Cardiovascular disease
ED: Emergency department
ESRD: End-stage renal damage
GFR: Glomerular filtration rate
IQR: Interquartile range
MACEs: Major adverse cardiac events
MicroRNA-1: MiR-1
MicroRNAs: MiRs
NPV: Negative predictive value
PE: Pulmonary embolism
PPCI: Primary percutaneous coronary intervention
PPV: Positive predictive value
qRT-PCR: Quantitative reverse transcriptase-polymerase chain reaction
ROC: Receiver operating characteristic curve
Sen.: Sensitivity(%)
Spe.: Specificity(%)
LR +: Positive likelihood ratio
LR -: Negative likelihood ratio
GRACE: Global acute coronary event registration score
REMS: Emergency medical score
